# Real-Time Medical Record Quality Control System (MRQCS): Development and Retrospective Longitudinal Observational Study

**DOI:** 10.2196/97456

**Published:** 2026-10-27

**Authors:** Shengrong Zhu, Honglin Shao, Chen Zhang, Wei Li, Xinye Xu, Yipei Wang, Hong Ji

**Affiliations:** 1 Department of Information Management and Big Data Center Peking University Third Hospital Beijing, Beijing China; 2 Department of Medical Affairs Peking University Third Hospital Beijing, Beijing China; 3 Department of Medical Affairs, Office of Internet Hospital Peking University Third Hospital Beijing, Beijing China

**Keywords:** medical record quality control, real-time prompt, block validation, defect detection, rule engine, AI-assisted quality control, interreviewer consistency

## Abstract

**Background:**

Medical record quality is a key focus of health care quality management. Traditional manual medical record quality control (QC) suffers from low efficiency, inability to achieve full-sample coverage, and poor consistency. Existing intelligent QC systems have significant deficiencies in real-time intervention and quantitative assessment of application effects.

**Objective:**

This study aimed to describe the design and development of the medical record quality control system (MRQCS) and to retrospectively evaluate its associations with medical record defect detection, medical record quality, and interreviewer consistency using 7-year longitudinal final-stage QC records (2019-2025).

**Methods:**

MRQCS implements a 2-level QC process for clinicians: nonblocking real-time prompting during documentation and blocking validation upon medical record completion. For QC reviewers, it provides AI-assisted defect detection and a 1-click write-back function. QC records from January 2019 to December 2025 were analyzed as 84 monthly discharge cohorts (43,324 sampled admissions), using interrupted time series (ITS) segmented regression with breakpoints aligned to the first discharge cohorts affected by each intervention (December 2020, July 2023, and May 2025). A stable‑rule‑set analysis was performed for genuine quality improvement. Linear mixed‑effects modeling with intraclass correlation coefficients (ICC) was applied to assess reviewer consistency, alongside multiple sensitivity analyses.

**Results:**

By 2025, the MRQCS QC rule library expanded from 41 rules initially to 597 rules. The sampled-case defect rate increased sharply after the January 2021 launch (trend change +0.06, 95% CI 0.03-0.08, *P*<.001), and rrates based on the 41 stable rules present in every year rose from 16.7% (880/5256) in 2021 to 50.9% (3662/7190) in 2023, indicating that most of the increase reflected improved system sensitivity. The high-risk case rate declined significantly after July 2023 (trend change –0.09, 95% CI –0.15 to –0.02, *P*=.007), and the residual defect rate showed an immediate decline at the May 2025 breakpoint (level change –0.44, 95% CI –0.85 to –0.03, *P*=.03). The AI-assisted write-back rate exceeded 97% after August 2023 and declined to 84%-87% after the May 2025 discharge blocking intervention (level change –2.14, 95% CI –3.08 to –1.21, *P*<.001), suggesting that clinicians corrected an increasing proportion of defects. Reviewer-level defect rates were far more homogeneous in the AI-assisted period (coefficient of variation [CV] 26.8% vs 115.7%), with reviewer identity accounting for a smaller share of the total variance (ICC 0.317 vs 0.782), indicating greater interreviewer consistency. Consistency of the high-risk defect rate showed the same pattern (CV 31.3% vs 135.3%; ICC 0.053 vs 0.541).

**Conclusions:**

Through the collaborative model of 2-level real-time QC and AI-assisted final-stage QC, MRQCS was associated with improved defect detection, reduction of documentation defects, and substantially greater interreviewer consistency; the discharge-time blocking intervention further reduced the residual defect rate. These findings provide a practical reference for the construction of medical record quality management systems.

## Introduction

Medical record quality control (QC) is a critical component of hospital health care quality management. The completeness, standardization, and accuracy of medical records are important indicators for measuring health care quality and management levels [1]. Numerous clinical studies use medical record documentation as the core data source for information extraction and predictive analysis [2-9], such as extracting subphenotypes of critical limb ischemia cases through improved natural language processing [2], predicting fall risk [3] and mortality [6] in older adult patients using clinical records, and identifying treatment-emergent adverse events [9]. Medical record quality directly determines the accuracy of research results and the scientific validity of clinical decision-making.

With the popularization of electronic health record (EHR) systems and the advancement of structured medical records, medical record quality has improved to some extent [10-14]. However, medical institutions both domestically and internationally still face issues with inconsistent medical record quality: a 2019 study on rural medical institutions in China showed serious deficiencies in basic diagnostic and treatment information in outpatient medical records [15]; a 2022 study at 2 Dutch medical centers indicated that even with standardized documentation, medical record quality remained at a low level [13], highlighting the urgent need for medical record quality improvement in health care institutions.

Quality assessment is the foundation of medical record quality improvement. Existing assessment tools such as QNOTE [16] and the Physician Documentation Quality Instrument [17,18] all rely on manual evaluation, which is labor-intensive and cannot achieve real-time assessment. Traditional manual QC adopts methods such as process control, 3-level QC, and 2-level random blind review, which are limited to sampling inspection and cannot achieve full-sample validation, consuming considerable time and effort. The development of AI and big data technologies has promoted research on AI-assisted medical record content QC [19,20]. Related systems achieve full-sample automatic QC through natural language processing for medical record structuring combined with authoritative standards for rule library construction. However, significant shortcomings remain: QC interventions are mostly triggered at scheduled times or specific nodes without achieving true real-time capability [19]; application effect assessments lack specificity and do not adequately reflect the system’s defect detection capabilities [20]; quantitative research on interreviewer consistency has not been conducted; QC result notification mechanisms are unclear, with excessive prompts interfering with clinical work and insufficient prompts leading to delayed defect handling.

In recent years, large language models (LLMs) such as ChatGPT (OpenAI) and DeepSeek (Hangzhou DeepSeek Artificial Intelligence Basic Technology Research Co Ltd) have shown promising applications in the health care field [21-23], with advantages in semantic understanding and complex defect identification that can provide new technical support for medical record QC. To address the above challenges, this study designed and developed a medical record quality control system (MRQCS) primarily based on rule engines and supplemented by LLMs and deeply integrated with clinical workflows, achieving nonblocking real-time prompts and blocking validation (defined as a mandatory validation step that prevents submission until flagged issues are deleted or resolved) for clinicians and AI-assisted defect detection with 1-click write-back for QC reviewers.

The objective of this study was to describe the design and development of MRQCS for clinicians and QC reviewers and to retrospectively evaluate its associations with medical record defect detection, medical record quality, and interreviewer consistency using 7 years of longitudinal final-stage QC records (2019-2025).

## Methods

### Study Design and Setting

This was a single-center, retrospective longitudinal evaluation of MRQCS implemented at a tertiary hospital in Beijing, China. The study evaluated the associations of MRQCS with defect detection, medical record quality, and interreviewer consistency. The system integrates multisource structured and unstructured in-hospital data; it provides real-time prompts and blocking validation for clinicians and offers AI-assisted defect detection and 1-click write-back for final QC reviewers. This evaluation included all final QC records of patients discharged between January 2019 and December 2025, with patient discharge month set as the temporal analysis unit, since QC is conducted on medical records after patient discharge.

### System Architecture and Workflow

The MRQCS proposed in this study adopts a 4-tier hierarchical architecture. From bottom to top, it consists of the data source layer, data collection layer, QC layer, and presentation layer, as shown in Figure 1.

**Figure 1:**
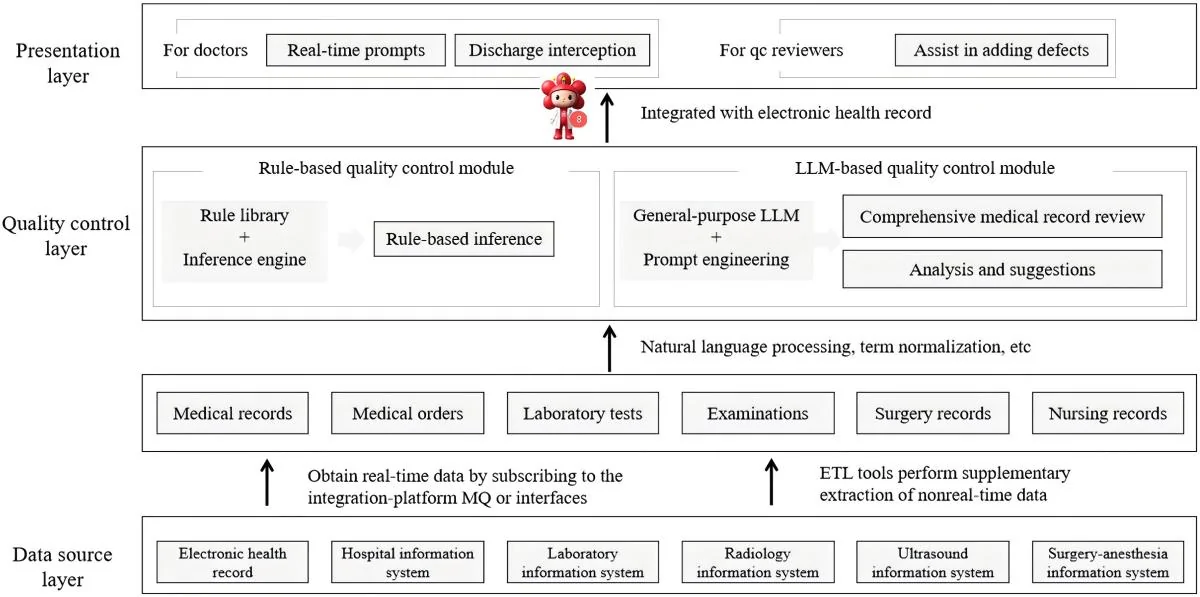
Four-tier architecture of MRQCS. ETL: extract, transform, load; LLM: large language model; MQ: message queue; MRQCS: medical record quality control system; QC: quality control.

### Data Source Layer

As the fundamental data foundation of the entire system, the data source layer connects to multiple core hospital business information systems, including the EHR system, hospital information system, laboratory information system, radiology information system, etc. This layer provides multidimensional raw clinical data, serving as the data source for upstream data collection modules.

### Data Collection Layer

The data collection layer is responsible for acquiring and processing multisource clinical data. It captures real-time streaming clinical data by subscribing to message queues of the hospital integration platform or via application programming interfaces. Meanwhile, extract, transform, load (ETL) tools are used to supplement the extraction of historical and offline nonreal-time data, balancing the timeliness and completeness of data acquisition. This layer processes both structured data (eg, laboratory reports, nursing vital signs, and physician order entries) and unstructured data (eg, textual diagnostic content in examination reports). The system leverages a BiLSTM-CRF (bidirectional long short-term memory–conditional random field) model [24-26] combined with domain-specific business rules for natural language processing. Raw collected data go through processing workflows, including word segmentation and term normalization, to complete text cleansing and medical term standardization. The processed data are then forwarded to the QC layer for subsequent analysis.

### QC Layer

#### Overview

As the core operational processing unit of the system, the QC layer consists of 2 submodules: the rule-based QC module and the LLM-based QC module.

#### Rule-Based QC Module

This module comprises a rule library and a rule-based inference engine. The rule library is built in accordance with national industry standards, including the Specification for Medical Record Documentation and the Quality Scoring Standard for Inpatient Medical Records, as well as the hospital’s internal medical record quality management regulations. QC rules are formulated and reviewed collaboratively by the hospital’s medical affairs department and medical record quality experts. The quality assessment covers 25 types of medical documents categorized into 8 main groups: progress notes, discharge or death records, admission notes, other medical records, medical record front page (the standardized summary form of each inpatient record, which serves as the primary data source for diagnosis-related groups [DRGs]–based payment and hospital performance evaluation in China), perioperative records, physician order sheets, and informed consent forms. Evaluation dimensions include document completeness, cross-source data consistency, and documentation timeliness. The rule library supports multiattribute configuration to define deduction items, risk levels (low, moderate, and high), and interception flags. Interception flags adjust the execution intensity of QC workflows to enable differentiated QC strategies: for example, missing surgical informed consent is configured as an interception rule, while nonstandard progress note documentation is set as a general alert rule. By conducting logical matching between case data and the predefined rule library, the inference engine identifies defects in medical records and returns standardized QC outputs through application interfaces. The returned outputs contain defect details, deduction scores, and risk levels, providing support for front-end QC alerts and defect resolution.

#### LLM-Based QC Module

This module is built on the on-premises LLMs DeepSeek-R1 and Qwen3-235B-A22B and is entirely deployed within the hospital’s secure internal network to ensure data privacy and compliance with the institution’s information security policies. Qwen3 is used as the default model to balance response latency and computational cost. Before clinical data is fed into the LLM, patient-identifiable information is removed, with only the clinical content required for analytical tasks retained. This module implements 2 core functions. **First, the Medical Record Review function** analyzes completed medical documents via prompt engineering and clinical context; it can identify potential medical record defects that are not captured by the rule library, such as missing key laboratory and examination findings in discharge summaries. The relevant prompts for this function are provided in Multimedia Appendix 1**. Second, the analysis and suggestions function** generates targeted revision suggestions based on the medical record context for defects detected by the rule-based module to assist clinicians in revising medical records, and the corresponding prompts for this function are also included in Multimedia Appendix 2. Limited by the concurrency capacity of locally deployed large models, LLM-based QC functions require manual triggering by clinicians. Outputs are returned approximately 1 second after activation, and fully automatic real-time concurrent QC is not currently supported.

### Presentation Layer

#### Overview

The presentation layer supports result visualization and human-computer interaction. Through deep integration with the EHR system, it delivers differentiated QC services to both clinicians and QC reviewers.

#### Integration With the EHR

The MRQCS achieves seamless end-to-end integration with the EHR system without disrupting routine clinical workflows. Key integration points are outlined in the following paragraphs.

**Regarding the EHR startup**, when the EHR system launches, the MRQCS activates automatically and appears as a floating widget in the bottom-right corner of the EHR interface. It does not occupy primary screen space and leaves normal clinical documentation operations unaffected.

**Regarding medical record saving**, when clinicians save or submit medical records within the EHR, data is asynchronously pushed to the MRQCS. The MRQCS performs multidimensional quality assessment using the associated clinical datasets and refreshes QC outputs asynchronously without interrupting ongoing documentation work.

**Regarding the medical record completion**, after a patient is discharged, clinicians trigger the “complete medical record” function within the EHR, which invokes interception validation from the MRQCS. If any interception rule is violated, a pop-up window displaying defect details will appear within the EHR, and clinicians must resolve or delete all intercepted defects before they can submit the record. A 5-second time-out mechanism is also implemented: if the MRQCS malfunctions, the EHR will continue its workflow to maintain clinical continuity.

**Regarding the final-stage QC**, QC reviewers retrieve records pending quality assessment through the EHR-embedded QC module. The MRQCS dynamically visualizes deduction scores and defect distribution for the retrieved records. When accessing the details of an individual record, reviewers can view all detected defects and write validated defects into the defect database with a single click.

### Two-Tier Prompt Mechanism for Clinical End Users

#### Stage 1: Nonblocking Real-Time Prompting During Documentation

When medical records are saved to the EHR, the floating MRQCS widget in the bottom-right corner displays the number of detected defects. Clicking the widget displays detailed defect information, including defect descriptions, deduction values, and risk levels (Figure 2). This notification workflow does not disrupt normal EHR operations. After clinicians revise and resave the documents, QC outputs are refreshed to form a closed-loop workflow covering documentation, quality review, and revision. A supplementary LLM-based review function is also available: after opening a target document, clinicians can activate the medical record review function, which identifies latent defects missed by rule-based checks and provides supporting rationales and revision guidance. For specific defects identified by rule-based QC, the analysis and suggestion function generates customized modification recommendations for the document.

**Figure 2:**
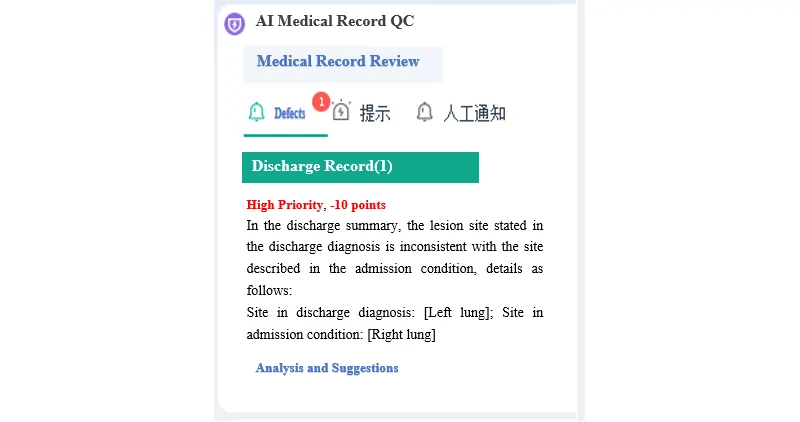
Real-time defect alerts during medical record documentation. QC: quality control.

#### Stage 2: Blocking Validation Upon Medical Record Completion

After a patient is discharged, mandatory blocking validation is triggered when clinicians initiate the “complete medical record” action. If interception rules are violated (eg, missing surgical informed consent documents), a mandatory pop-up window will appear in the EHR, requiring clinicians to revise the relevant documents. Manual removal of false-positive defects is also supported. This process is shown in Figure 3.

**Figure 3:**
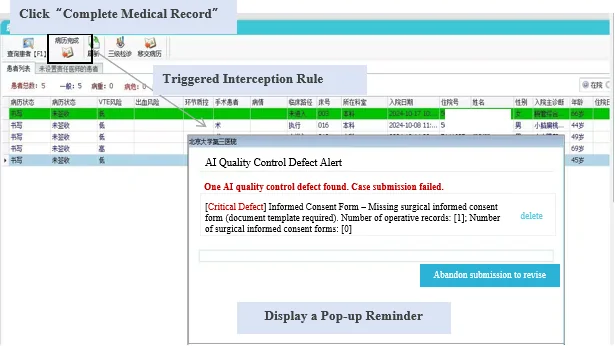
Blocking validation pop-up upon medical record completion.

#### AI-Assisted Defect Addition for the QC Reviewers

For monthly retrospective record audits, high defect scores exported from MRQCS are combined with randomly sampled cases and assigned to quality reviewers. When reviewers confirm the system-identified true-positive defects, they can use the “write-back” function to directly import validated defects to the QC database (Figure 4). False-positive results can be deleted manually. Reviewers may also manually add new defects missed by the system, enabling complementary collaboration between AI-assisted QC and manual record review.

**Figure 4:**
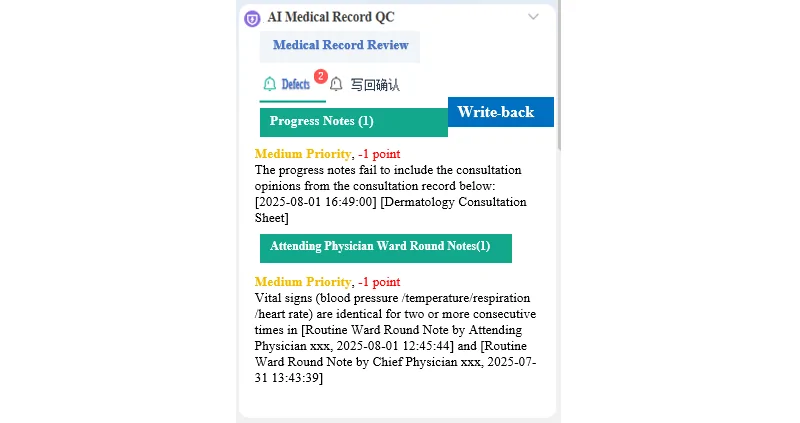
AI-assisted defect addition for the QC reviewers. QC: quality control.

### Data Source and Study Population

This evaluation included all QC records of patients discharged between January 2019 and December 2025 stored in the MRQCS QC database. Each record corresponds to 1 QC entry for 1 inpatient admission: defect cases have 1 record per detected defect, while defect-free sampled cases are represented by a single record with empty defect fields. After deidentification, 61,697 QC records from 47 QC reviewers were retrieved; 53 records from 28 reviewers with fewer than 10 records were excluded, leaving 61,644 records from 19 reviewers for analysis. The included records were reconstructed into case-level observations, and the final analytic sample covered 43,324 inpatient admissions (defined by the unique combination of patient identifier and discharge date-time) that received monthly sampling QC during the study period (Table 1). Discharge date-times span January 2019 to December 2025 with no missing values, forming 84 monthly discharge cohorts. For the reviewer consistency analysis, joint-reviewer cases (case defects attributed to more than 1 reviewer) were further excluded, and only reviewers with a substantial workload (≥100 cases reviewed within each study period) were retained (6 reviewers in each of the manual and AI-assisted periods, with 127 and 144 reviewer-month observations, respectively; Figure 5). Table 1 summarizes the annual numbers of sampled cases, defect cases, high-risk cases, defect records, and AI-assisted write-back records across the 7-year study period.

**Table 1:** Annual summary of sampled cases, defect cases, high-risk cases, defect records, and AI-assisted write-back volume, 2019-2025.

Year	Sampled cases, n	Defect cases, n (%)	High-risk cases, n (%)	Defect records, n	AI-assisted write-back, n
2019	6491	1327 (20.4)	74 (1.14)	1706	0
2020	4888	995 (20.4)	114 (2.33)	1239	0
2021	5256	1123 (21.4)	134 (2.55)	1594	198
2022	5875	2400 (40.9)	276 (4.70)	4477	1149
2023	7190	4229 (58.8)	514 (7.15)	9735	7814
2024	6290	3877 (61.6)	619 (9.84)	7566	7432
2025	7334	4401 (60.0)	326 (4.45)	8955	8221
Total	43,324	18,352 (42.36)	2057 (4.75)	35,272	24,814

**Figure 5:**
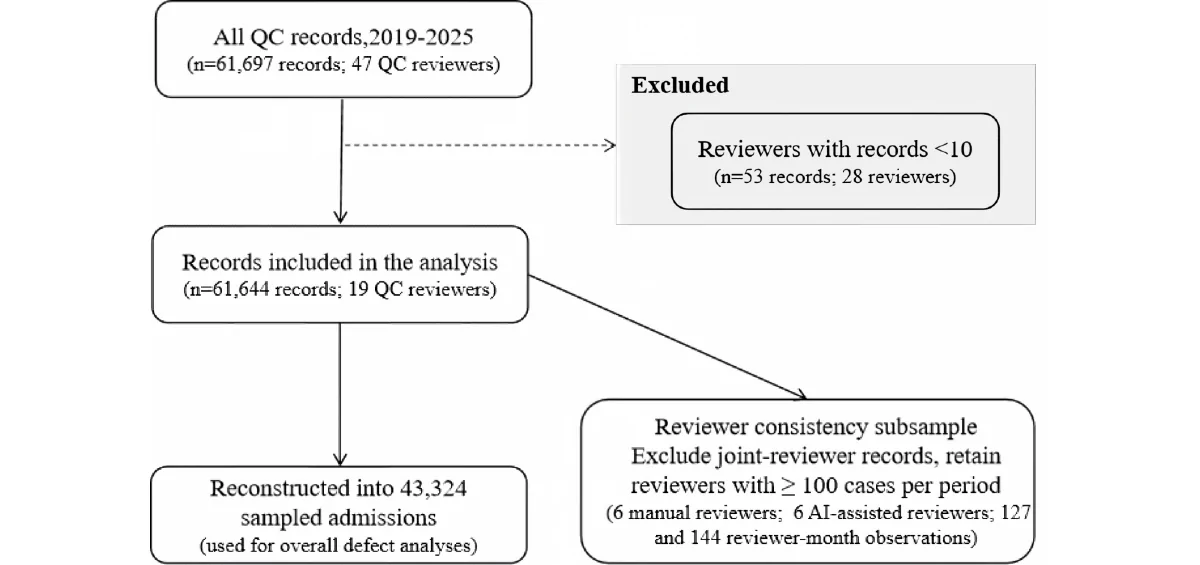
Participant flow diagram of quality control records, 2019-2025. QC: quality control.

### Sampling Procedure and Case Definitions

Monthly sampling QC was conducted by trained QC reviewers. Before system deployment, reviewers manually selected cases stratified by department for verification. After MRQCS implementation, the Medical Affairs Department assigned review tasks by combining high-deduction records exported from MRQCS with randomly sampled admissions. It should be noted that our dataset lacks metadata to distinguish between records from high-deduction lists and those from pure random sampling, which may introduce residual selection bias between the preimplementation and postimplementation periods. A case is classified as a defect case if it contains at least one defect record and as a defect-free sampled case if the admission is represented by a single record with empty defect fields. A high-risk case is defined as any case with at least one defect score ≥ 10. The AI field indicates whether a defect was added via the AI-assisted write-back workflow (1=AI-assisted, 0=manually added; empty for defect-free sampled cases).

### Intervention Timeline

MRQCS was rolled out in phases. In January 2021, its real-time clinical QC and final-stage QC assistance functions were launched, with 41 predefined rules configured. In June 2021, the Medical Affairs Department recommended the system to reviewers, and it was approved as an auxiliary tool. In August 2023, with 335 rules in total, final-stage QC transitioned to the mode of “AI pre-verification as primary, manual review as supplementary.” A discharge-blocking function with 597 rules was deployed in May 2025. Final QC was typically conducted around 1 month after patient discharge (eg, QC for discharge cases from August is performed at the end of September to early October), so each intervention was assigned to the first discharge cohort affected by the corresponding measure. However, the discharge-blocking function operates in real time. Accordingly, the month when the function was activated was defined as the intervention time point: December 2020 (B1), July 2023 (B2), and May 2025 (B3).

### Outcome Measures

Four monthly outcome indicators were analyzed as follows: (1) sampled-case defect rate (number of defect cases / number of sampled cases × 100%), (2) high-risk case rate (number of cases with at least one defect score ≥ 10 / the number of sampled cases × 100%), (3) average number of defects per sampled case, and (4) AI-assisted defect write-back rate (number of AI-assisted defect records / the total number of defect records × 100%), which reflects the proportion of documented defects entered via the AI-assisted workflow. We additionally examined the type-specific detection rate across 8 document categories.

### Statistical Analysis

We conducted the primary analysis using interrupted time series (ITS) segmented regression on data from 84 monthly discharge cohorts. For the defect rate, high-risk rate, and AI write-back rate, we fitted quasi-binomial logistic regression models (on the logit scale) with the number of sampled cases set as the binomial denominator; for the mean defect count, we fitted a Poisson model with a log link. Each model included a continuous time term, 3 level-change indicators (D), and 3 trend-change terms (T) corresponding to the 3 breakpoints: December 2020, July 2023, and May 2025. All models were estimated with heteroskedasticity-robust SEs. We computed counterfactual predictions on the logit scale by extending prebreakpoint trends, then transformed these predictions back to the probability scale to compare observed rates with the values expected if none of the interventions had been implemented. To distinguish improved detection capability from genuine quality improvement, we repeated the ITS analysis on a stable rule set consisting of the 41 defect codes that appeared in every year from 2019 to 2025. Finally, we assessed residual autocorrelation using the Durbin-Watson statistic and the Ljung-Box test.

We evaluated reviewer consistency after excluding joint-reviewer records and restricting the analysis to reviewers who reviewed at least 100 cases in each study period, which retained 6 reviewers in both the manual review period and the AI-assisted review period. For each period, we fitted a linear mixed-effects model with a random reviewer intercept (adjusted for time) to the reviewer-month mean defect counts and high-risk defect rates (defined as cases with at least one defect score ≥ 10). We calculated the intraclass correlation coefficient (ICC) as the ratio of reviewer variance to total variance and tested reviewer effects using likelihood-ratio tests. We additionally summarized between-reviewer dispersion with the coefficient of variation (CV) of reviewer-level defect rates.

Sensitivity analyses involved shifting each breakpoint by ±1 and ±2 months, excluding 2020 (the COVID-19 pandemic period), adding a placebo breakpoint set at January 2020, and testing an alternative high-risk threshold of 5. For the reviewer consistency analysis, we additionally tested alternative workload thresholds (≥50, ≥200, and ≥500 cases per period). All statistical tests were 2-sided, with the significance level set at 0.05. All analyses were performed in Python (version 3.12, Python Software Foundation) using the Statsmodels (version 0.15), SciPy, and pandas libraries.

### Ethical Considerations

This retrospective study was approved by the Medical Science Research Ethics Committee of Peking University Third Hospital (IRB00006761-M20250619). It did not involve the collection of personal privacy data, strictly adhering to the Declaration of Helsinki. Given the retrospective nature of the study and the use of deidentified data, the requirement for informed consent was waived.

### Reporting Guideline

This study was reported in accordance with the STROBE (Strengthening the Reporting of Observational Studies in Epidemiology) statement for observational studies, and the completed STROBE checklist is provided in Multimedia Appendix 3.

## Results

### ITS Analyses

Table 2 presents the ITS regression coefficients for all outcomes on the logit scale, using quasi-binomial logistic models with heteroskedasticity-robust SEs. Outcome Y3 was fitted with a Poisson model with a log link. All *P* values are 2-sided, where “—” indicates the result is not estimable. Since the AI-assisted write-back rate was 0 before January 2021, the model for Y4 was fitted using data from January 2021 onward.

**Table 2:** Interrupted time series regression coefficients on the logit scale.

Coefficient and values		Y1 defect rate	Y2 high-risk rate	Y3 mean defects per case	Y4 AI write-back rate	Stable rule set
Intercept						
	β with 95% CI	–1.39 (–1.59 to –1.20)	–4.87 (–5.28 to –4.45)	–1.33 (–1.54 to –1.12)	–4.22 (–7.82 to –0.61)	–1.66 (–1.86 to –1.46)
	*P* value	<.001	<.001	<.001	.02	<.001
Time						
	β with 95% CI	0.00 (–0.01 to 0.02)	0.06 (0.03 to 0.09)	–0.00 (–0.02 to 0.01)	0.17 (0.00 to 0.34)	0.00 (–0.01 to 0.02)
	*P* value	.59	<.001	.93	.046	.73
Level change December 2020 (B1)						
	β with 95% CI	–0.33 (–0.67 to 0.01)	–0.42 (–1.25 to 0.40)	–0.27 (–1.10 to 0.55)	—^a^	–0.37 (–0.71 to –0.03)
	*P* value	.06	.32	.52	—	.03
Trend change December 2020 (B1)						
	β with 95% CI	0.06 (0.03 to 0.08)	–0.02 (–0.07 to 0.02)	0.07 (0.01 to 0.13)	—	0.06 (0.04 to 0.08)
	*P* value	<.001	.32	.02	—	<.001
Level change July 2023 (B2)						
	β with 95% CI	0.50 (0.02 to 0.97)	0.78 (–0.17 to 1.73)	–0.28 (–1.28 to 0.72)	2.50 (0.39 to 4.61)	0.37 (–0.07 to 0.80)
	*P* value	.04	.11	.59	.02	.10
Trend change July 2023 (B2)						
	β with 95% CI	–0.07 (–0.10 to –0.04)	–0.09 (–0.15 to –0.02)	–0.07 (–0.12 to –0.01)	–0.14 (–0.31 to 0.04)	–0.06 (–0.09 to –0.03)
	*P* value	<.001	.007	.03	.12	<.001
Level change May 2025 (B3)						
	β with 95% CI	–0.44 (–0.85 to –0.03)	–0.48 (–1.03 to 0.07)	–0.34 (–0.60 to –0.07)	–2.14 (–3.08 to –1.21)	–0.40 (–0.79 to –0.02)
	*P* value	.03	.09	.01	<.001	.04
Trend change May 2025 (B3)						
	β with 95% CI	0.10 (0.05 to 0.14)	0.19 (0.12 to 0.26)	0.05 (0.02 to 0.09)	–0.10 (–0.26 to 0.06)	0.08 (0.04 to 0.12)
	*P* value	<.001	<.001	.002	.23	<.001

^a^Not available.

### Overall Defect Rate (Y1)

Table 2 and Figure 6 show that the overall sampled-case defect rate remained stable before 2021 (time trend 0.00, 95% CI –0.01 to 0.02, *P*=.59), and increased sharply after the launch in January 2021 (trend change 0.06, 95% CI 0.03-0.08, *P*<.001). This was followed by a further level increase at the July 2023 breakpoint (level change 0.50, 95% CI 0.02-0.97, *P*=.04), alongside a subsequent declining trend (trend change –0.07, 95% CI –0.10 to –0.04, *P*<.001). At the May 2025 breakpoint, the defect rate saw a significant immediate decline (level change –0.44, 95% CI –0.85 to –0.03, *P*=.03), followed by an upward trend (trend change 0.10, 95% CI 0.05-0.14, *P*<.001). Counterfactual analysis estimated that, in absolute percentage-point terms, the sampled-case defect rate was 0.44 higher by August 2023 and 0.38 higher by December 2025 compared with the no-intervention counterfactual (Figure 6). This finding is consistent with a substantial expansion of the system detection capacity.

**Figure 6:**
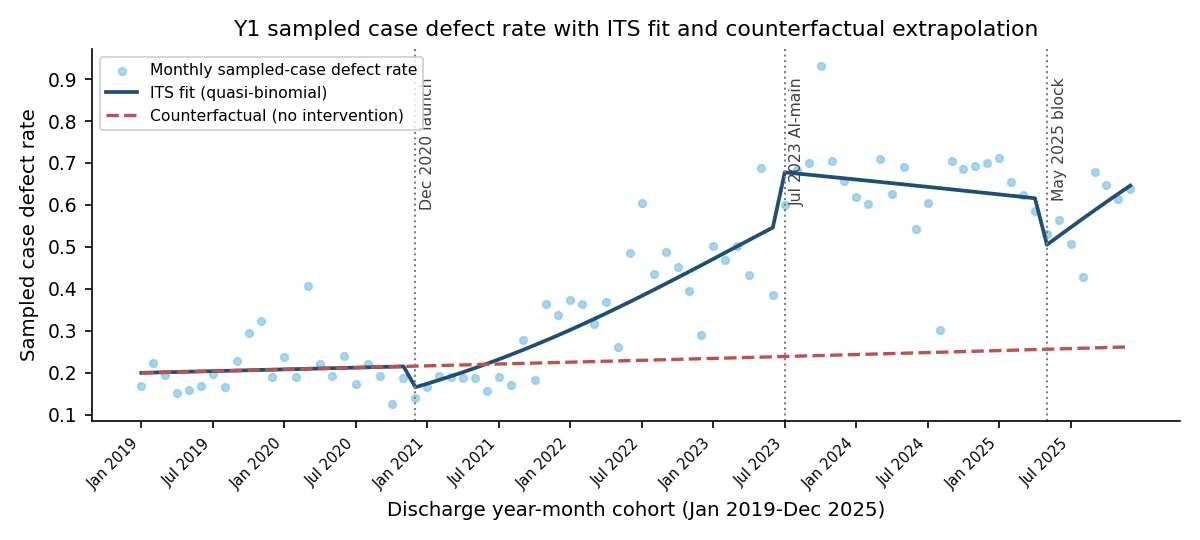
Monthly sampled-case defect rate with ITS fitting, breakpoints (December 2020, July 2023, May 2025), and counterfactual extrapolation assuming no interventions. ITS: interrupted time series.

### High-Risk Case Rate (Y2)

Table 2 and Figure 7 show that the high-risk case rate increased steadily before 2023 (time trend 0.06, 95% CI 0.03-0.09, *P*<.001) and declined significantly after the July 2023 breakpoint (trend change –0.09, 95% CI –0.15 to –0.02, *P*=.007), with a further level reduction at the May 2025 breakpoint (–0.48, 95% CI –1.03 to 0.07; *P*=.09) and a strong upward trend thereafter (trend change 0.19, 95% CI 0.12-0.26, *P*<.001). Counterfactual analysis estimated that, compared with the preintervention trend, the high-risk case rate was 0.42 lower by May 2025 and 0.48 lower by December 2025, indicating a genuine reduction of high-risk defects.

**Figure 7:**
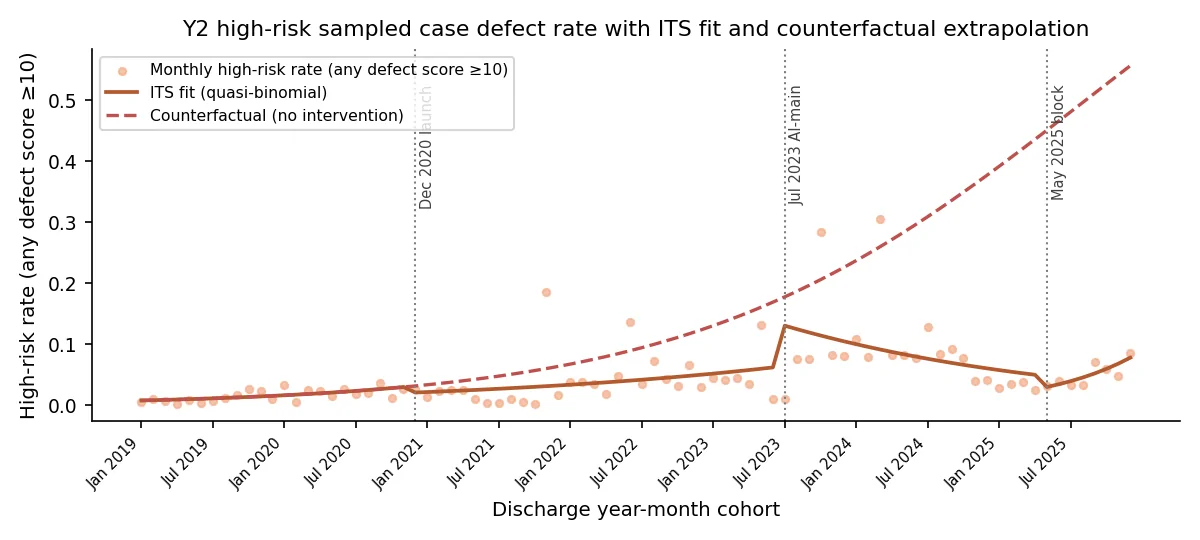
Monthly high-risk case rate (proportion with 1 defect score ≥ 10) with ITS fit and counterfactual extrapolation assuming no interventions. ITS: interrupted time series.

### AI-Assisted Defect Write-Back Rate (Y4)

Table 2 and Figure 8 show that the AI-assisted write-back rate was 0% in 2019-2020 and increased after the system launch, reaching a plateau above 97% after the August 2023 shift to AI-primary review (level change 2.50, 95% CI 0.39-4.61, *P*=.02). After the May 2025 discharge blocking intervention, the write-back rate declined significantly (level change –2.14, 95% CI –3.08 to –1.21, *P*<.001), stabilizing at 84%-87%. This is consistent with the trend that clinicians correct an increasing proportion of defects during hospitalization through real-time prompts and blocking validation (Figure 8).

**Figure 8:**
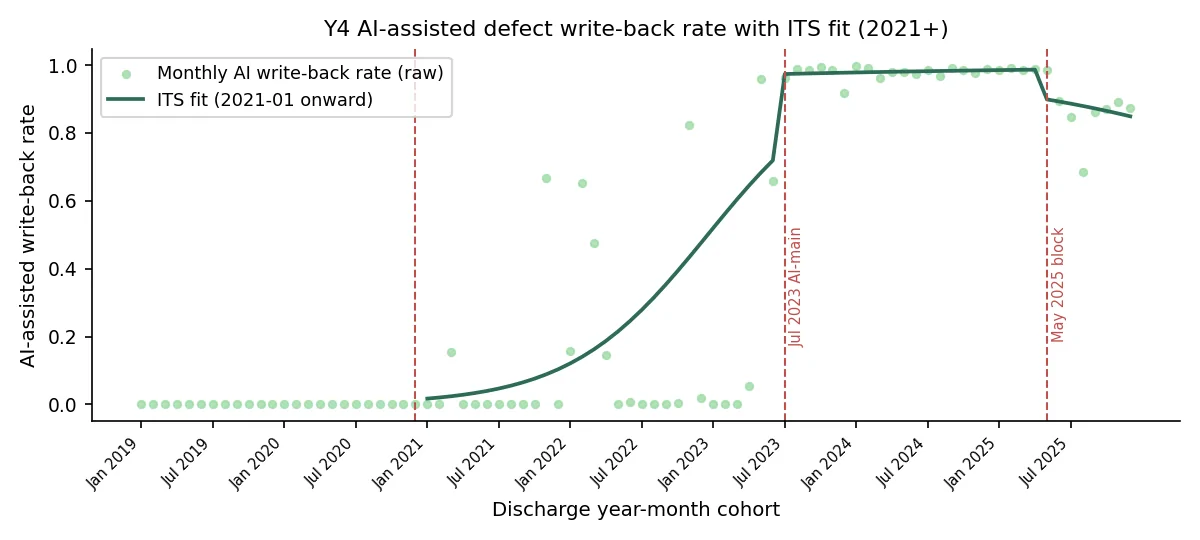
Monthly AI-assisted defect write-back rate with ITS fit (January 2021 onward). ITS: interrupted time series.

### Stable-Rule-Set Analysis

To distinguish improved detection from genuine quality improvement, we repeated the ITS analysis for the 41 defect codes included in every year from 2019 to 2025, holding the rule set constant (Figure 9 and Table 2). The stable-rule defect rate rose from 16.7% (880/5256) in 2021 to 50.9% (3662/7190) in 2023, and declined significantly after July 2023 (trend change –0.06, 95% CI –0.09 to –0.03, *P*<.001) and in May 2025 (level change –0.40, 95% CI –0.79 to –0.02, *P*=.04). Since the rule set was fixed, the increase before 2023 reflects higher detection intensity associated with AI-assisted review, while the subsequent decline indicates genuine quality improvement across stable dimensions. The gap between the all-rules curve and the stable-rules curve remained modest (5%-8% in 2023-2025). At the case level, the stable rules alone identified 86.1% (3338/3877) to 92.8% (4086/4401) of all defective cases, which indicates that rule library expansion added only a limited number of new defective cases. Therefore, the overall increase in defect rate was driven predominantly by improved detection sensitivity rather than rule growth. At the record level, however, rules not present in every year accounted for 16.3% (1464/8955) to 27.2% (2646/9735) of defect records during 2023-2025, indicating that newly added rules contributed additional defect types within already-flagged cases.

**Figure 9:**
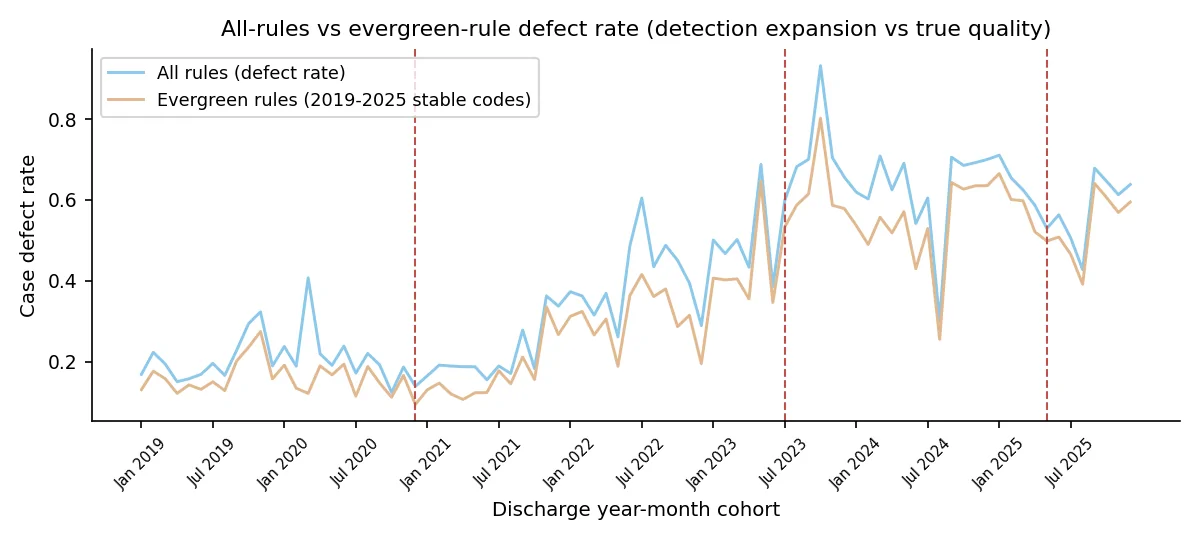
Monthly defect rates based on the full rule set vs the stable 41-code rule set.

### Mean Number of Defects per Case (Y3)

The mean number of defects per sampled case increased after January 2021 (trend change 0.07, 95% CI 0.01-0.13, *P*=.02), declined after July 2023 (trend change –0.07, 95% CI –0.12 to –0.01, *P*=.02), and showed a significant immediate reduction in May 2025 (level change –0.34, 95% CI –0.60 to –0.07, *P*=.01) followed by a subsequent upward trend (trend change 0.05, 95% CI 0.02-0.08, *P*=.002).

### Reviewer Consistency

To limit the influence of reviewers with negligible workloads on consistency estimates, we restricted the analysis to reviewers who completed at least 100 reviews within each period after excluding joint-reviewer records. This left 6 reviewers in each period: there were 127 reviewer-month observations in the manual period and 144 in the AI-assisted period; reviewers completed a median of 1926 cases (IQR 813-2997) in the manual period and 2,255 cases (IQR 2229-2297) in the AI-assisted period. Reviewer-level defect rates varied widely in the manual period (mean 0.56, SD 0.64, CV 115.7%), but were far more homogeneous in the AI-assisted period (mean 1.22, SD 0.33, CV 26.8%). In linear mixed-effects models with a random reviewer intercept, adjusted for time, reviewer identity accounted for 78.2% of the total variance in the manual period (ICC 0.782; reviewer variance 0.244 vs residual variance 0.068; likelihood-ratio test *P*<.001), but only 31.7% in the AI-assisted period (ICC 0.317; reviewer variance 0.100 vs residual variance 0.215; *P*<.001; Table 3). The period-by-time interaction was not statistically significant (*P*=.28), confirming that the reduction in reviewer identity variance was not attributable to differential time trends between the 2 periods; systematic differences between reviewers were thus substantially smaller when AI assistance was the primary workflow.

**Table 3:** Reviewer consistency for the overall and high-risk defect rates by period.

Outcome and period		Reviewers, n	Observations, n	Rate, mean (SD)	CV^a^, %	ICC^b^	Reviewer effect LRT^c^ *P* value
Overall defect rate							
	Manual (2019-2020)	6	127	0.56 (0.64)	115.7	0.782	<.001
	AI-assisted (2024-2025)	6	144	1.22 (0.33)	26.8	0.317	<.001
High-risk defect rate							
	Manual (2019-2020)	6	127	0.034 (0.046)	135.3	0.541	<.001
	AI-assisted (2024-2025)	6	144	0.070 (0.022)	31.3	0.053	.26

^a^CV: coefficient of variation.

^b^ICC: intraclass correlation coefficient.

^c^LRT: likelihood-ratio test.

Consistency of the high-risk defect rate showed a similar pattern (Table 3): reviewer-level rates ranged from 0.13% to 12.07% in the manual period (mean 3.4%, SD 4.6%, CV 135.3%), but converged to the range of 2.9%-8.8% in the AI-assisted period (mean 7%, SD 2.2%, CV 31.3%). The ICC declined from 0.541 (reviewer variance 0.0185 vs residual variance 0.0157; likelihood-ratio test *P*<.001) to 0.053 (reviewer variance 0.0003 vs residual variance 0.0057; *P*=.26), which indicates that when AI assistance is adopted as the primary workflow, systematic differences between reviewers in detecting high-risk defects are no longer detectable.

### Document-Type-Specific Detection Rates

Comparisons across document types are purely descriptive. Because aggregated document-type rates do not include individual-level case identifiers, formal hypothesis testing and normality-based selection between parametric and nonparametric statistical tests could not be performed. Table 4 presents defect detection rates for the 8 document types in 2019-2020 vs 2024-2025. Detection rates rose considerably for progress notes (+597.9%), discharge or death records (+516.1%), admission records (+280.5%), and medical record front pages (+278.7%), while the rate for other records dropped by 85.8%. The decrease in detection rates for certain document types under AI-assisted review can be explained by 3 categories of defects. First, complex semantic and logical defects, such as unclear stratification of the present illness history or disordered narrative chronology, were detected less often. This is because the current rule library can only identify explicit logical errors (eg, “the duration of time-related items in the present illness history exceeds the patient's age”) and has limited coverage of underlying semantic issues. Second, defects without corresponding QC rules, such as missing assessment forms in other records, cannot be automatically recognized, as they have not been added to the rule library, resulting in a clear drop in detection. Third, simple omission defects, such as blank or incorrect entries in the drug allergy field, can be corrected in real time by clinicians during documentation when prompted by AI. Additionally, analysis of defects manually added by QC reviewers in 2025 showed that all of these defects belonged to types not covered by the MRQCS rule library, which confirms that there are still blind spots in current defect detection and the rule library needs further improvement.

**Table 4:** Defect detection rates by document type before (2019-2020) and after (2024-2025) AI assistance.

Document type	Before (2019-2020, %)	After (2024-2025, %)	Relative change (%)
Admission records	9.4	35.8	+280.5
Discharge or death records	1.7	10.7	+516.1
Medical record front pages	2.3	8.9	+278.7
Informed consent forms	1.0	0.7	–33.1
Other records	0.3	0.0	–85.8
Perioperative records	2.8	8.1	+190.6
Physician orders	0.0	0.1	+101.7
Progress notes	8.2	57.0	+597.9

### Sensitivity Analyses

The results of the ITS analysis remained robust across all sensitivity analyses (Table 5): shifting the July 2023 breakpoint by ±1 or ±2 months produced level changes between 0.19 and 0.52, and slope changes between –0.057 and –0.088, all with the same direction of effect. Excluding data from 2020 (the COVID-19 pandemic period) generated consistent estimates of 0.50 for the level change and –0.072 for slope change. Setting a placebo breakpoint at January 2020 detected no significant effect (*P*=.98), which supports that the observed effect is not spurious. In addition, using an alternative high-risk threshold of 5 still yielded consistent conclusions. The Durbin-Watson statistic was 1.89, and the Ljung-Box test was not statistically significant (*P*=.35), indicating no substantial residual autocorrelation.

**Table 5:** Sensitivity analyses for the interrupted time series model of the defect rate (July 2023 breakpoint estimates).

Scenario	Level change at B2 (β)	Slope change at B2 (β)
Breakpoints –2 months	+0.39	–0.057
Breakpoints –1 month	+0.19	–0.058
Breakpoints +1 month	+0.52	–0.083
Breakpoints +2 months	+0.42	–0.088
Excluding 2020	+0.50	–0.072
Placebo breakpoint, January 2020	+0.01^a^	—^b^
High-risk threshold 5	+0.76	–0.084

^a^*P*=.98.

^b^Not available.

For reviewer consistency, sensitivity analyses using alternative workload thresholds (≥50, ≥200, and ≥500 cases per period) yielded consistent conclusions (Table 6): the AI-assisted ICC remained stable at 0.317, the manual ICC ranged from 0.549 (≥200 cases, 5 reviewers) to 0.782 (≥50-100 cases, 6 reviewers), and in all scenarios the AI-assisted period showed a smaller reviewer variance share and lower reviewer-level CV, indicating that the conclusion of greater interreviewer consistency with AI assistance was robust to the workload threshold.

**Table 6:** Sensitivity analysis of the ICCa to the reviewer workload threshold.

Threshold (cases per period)	Manual period: reviewers, n	Manual period: ICC	AI-assisted period: reviewers, n	AI-assisted period: ICC
≥50	6	0.782	6	0.317
≥100 (main analysis)	6	0.782	6	0.317
≥200	5	0.549	6	0.317
≥500	5	0.549	6	0.317

^a^ICC: intraclass correlation coefficient.

## Discussion

### Principal Findings

Based on the complete 84-month time series from 2019 to 2025, this study found that the deployment of MRQCS was associated with a significant expansion of defect detection, a clear reduction in high-risk documentation defects after July 2023, an immediate drop in the residual defect rate after the May 2025 discharge blocking intervention, and considerably higher interreviewer consistency during the AI-assisted period (reviewer-level CV 26.8% vs 115.7%; ICC 0.317 vs 0.782). Through the stable-rule-set analysis, we successfully distinguished detection expansion from actual quality improvement. These findings directly meet the 2 primary objectives of this study: describing the development and implementation of the system and retrospectively evaluating the system’s associations with defect detection, documentation quality, and interreviewer consistency.

### Interpretation and Comparison With the Literature

Compared with the scheduled or node-based QC systems described in the literature [19], the most distinctive feature of MRQCS is its in-process intervention capability: it delivers nonblocking real-time prompts to clinicians while they are writing medical records and enforces blocking validation when records are completed. This allows defects to be intercepted before they are added to the permanent medical record, rather than being detected days or weeks afterward. Meanwhile, the system maintains the efficiency of clinical workflow through asynchronous, nonblocking notifications and a 5-second time-out mechanism, which guarantees the continuity of clinical work even when the system malfunctions.

Regarding the quantitative evaluation of system effects, most existing studies on earlier AI-based MRQCSs either lacked rigorous effect assessments or only reported results with limited specificity [20]. By contrast, this study used a complete 7-year, 84-month time series and an ITS design with breakpoints aligned to the intervention timing. It applied counterfactual prediction, adopted a stable-rule-set analysis to distinguish improved detection capability from true quality improvement, and used linear mixed-effects models with ICCs to quantify interreviewer consistency.

### Limitations

This study has several limitations. First, because the dataset did not retain a sampling-source indicator (random sample vs high-deduction list), residual selection differences between study periods cannot be completely ruled out, though the stable-rule-set analysis and the high-risk defect rate help partially address this concern. Second, the accuracy of MRQCS defect detection was not directly evaluated; instead, the AI-assisted defect write-back rate was used to indirectly reflect system usability. This is because the manpower cost of a full manual review of all records is prohibitively high, and the process by which reviewers add defects via the system already includes a manual verification step that can, to a certain extent, reflect the accuracy of system detection. Third, the reviewer cohorts in the manual period and the AI-assisted period were not identical, and reviewers did not assess overlapping sets of medical records across the 2 periods. Despite mixed-effect adjustment and ICC quantification, observed changes in interreviewer consistency may still be confounded by differences in reviewer training, case-mix composition, and institutional QC policy updates. Fourth, due to the retrospective study design, confounding factors could not be fully controlled. Fifth, LLM-assisted QC has not yet realized automatic triggering, primarily due to constraints on system execution efficiency and hardware resource consumption; currently, it only serves as a supplementary function that is manually triggered by clinicians.

Based on the limitations outlined above, our future work will focus on three aspects: (1) further improving the comprehensiveness of the rule library to enhance the system’s defect detection capability; (2) leveraging LLMs and model fine-tuning to explore their detection performance for long-tail defects and defects with complex semantics [27-29], with a particular focus on improving the model’s response speed and detection precision; and (3) continuously tracking the number of medical record QC defects to provide more robust evidence for system optimization, clinical management, and the improvement of overall medical record quality.

### Conclusions and Implications

Using the complete 84-month time series of QC records spanning from 2019 to 2025, this study demonstrates that the deployment of a real-time AI-assisted MRQCS is associated with multiple notable improvements: a significant expansion in defect detection capability, a genuine reduction in high-risk documentation defects, higher interreviewer consistency during the AI-assisted period, and an immediate drop in the residual defect rate after the discharge blocking intervention implemented in May 2025. Beyond the scope of this single hospital, this study provides a replicable model for integrating rule-based and AI-assisted QC into clinical documentation workflows. In this model, real-time, full-sample in-process QC complements traditional post-hoc sampling QC, while graded interventions, including non-blocking prompts and discharge blocking, can be rolled out incrementally, with measurable effects at each stage. Future research should incorporate blinded reference-standard evaluation of detection accuracy, direct measurement of system efficiency, and multicenter validation.

## Appendix

Multimedia Appendix 1 Prompt for medical record review–admission record.

Multimedia Appendix 2 Prompt for analysis and suggestions.

Multimedia Appendix 3 STROBE checklist.

## Abbreviations

BiLSTM-CRF: bidirectional long short-term memory–conditional random field
CV: coefficient of variation
DRG: diagnosis-related group
EHR: electronic health record
ETL: extract, transform, load
ICC: intraclass correlation coefficient
ITS: interrupted time series
LLM: large language model
MRQCS: medical record quality control system
QC: quality control
STROBE: Strengthening the Reporting of Observational Studies in Epidemiology

## Data Availability

The datasets analyzed in this study are available from the corresponding author upon reasonable request.
