# Supplementary material for: Real-Time Medical Record Quality Control System (MRQCS): Development and Retrospective Longitudinal Observational Study
Source: JMIR Med Inform. 2026 Oct 27;14:e97456. doi: 10.2196/97456 (PMC13752590; doi:10.2196/97456)
Supplement: Supplementary file 3 [file medinform_v14i1e97456_app3.docx]

STROBE Statement—checklist of items that should be included in reports of observational studies

|  | Item No. | Recommendation | Page  No. | Relevant text from manuscript |
| --- | --- | --- | --- | --- |
| **Title and abstract** | 1 | (*a*) Indicate the study’s design with a commonly used term in the title or the abstract | p. 1 | Title: “Development and Retrospective Evaluation…” |
|  |  | (*b*) Provide in the abstract an informative and balanced summary of what was done and what was found | p. 1 | Structured abstract: Background / Objective / Methods / Results / Conclusion / Keywords |
| Introduction | | | |  |
| Background/rationale | 2 | Explain the scientific background and rationale for the investigation being reported | pp. 2–3 | Introduction, paragraphs 1–3 (QC importance; limitations of manual QC and existing systems) |
| Objectives | 3 | State specific objectives, including any prespecified hypotheses | p. 3; p. 1 | Objective statement (Introduction, last paragraph) and Abstract Objective |
| Methods | | | |  |
| Study design | 4 | Present key elements of study design early in the paper | p. 4 | Study Design and Setting: single-center, retrospective longitudinal evaluation |
| Setting | 5 | Describe the setting, locations, and relevant dates, including periods of recruitment, exposure, follow-up, and data collection | p. 4; p. 10 | Tertiary hospital in Beijing, China; discharges January 2019–December 2025; 84 monthly discharge cohorts |
| Participants | 6 | (*a*) *Cohort study*—Give the eligibility criteria, and the sources and methods of selection of participants. Describe methods of follow-up  *Case-control study*—Give the eligibility criteria, and the sources and methods of case ascertainment and control selection. Give the rationale for the choice of cases and controls  *Cross-sectional study*—Give the eligibility criteria, and the sources and methods of selection of participants | pp. 10–11 | Data Source and Study Population; Sampling Procedure and Case Definitions; Figure 5 flow diagram |
|  |  | (*b*) *Cohort study*—For matched studies, give matching criteria and number of exposed and unexposed  *Case-control study*—For matched studies, give matching criteria and the number of controls per case | - | - |
| Variables | 7 | Clearly define all outcomes, exposures, predictors, potential confounders, and effect modifiers. Give diagnostic criteria, if applicable | pp. 11–12 | Sampling Procedure and Case Definitions (defect case, defect-free sampled case, high-risk case); Outcome Measures (Y1–Y4) |
| Data sources/ measurement | 8* | For each variable of interest, give sources of data and details of methods of assessment (measurement). Describe comparability of assessment methods if there is more than one group | pp.10-12 | Data Source and Study Population ; Outcome Measures |
| Bias | 9 | Describe any efforts to address potential sources of bias | p. 11; pp. 23–24 | Sampling limitation acknowledged; stable-rule-set analysis; high-risk rate; Discussion Limitations |
| Study size | 10 | Explain how the study size was arrived at | pp. 10–11 | All final QC records in the study period (61,697 retrieved; 61,644 included; 43,324 admissions); |

Continued on next page

| Quantitative variables | 11 | Explain how quantitative variables were handled in the analyses. If applicable, describe which groupings were chosen and why | pp. 12–13 | Monthly discharge cohorts; rates defined in Outcome Measures; breakpoints from Intervention Timeline |
| --- | --- | --- | --- | --- |
| Statistical methods | 12 | (*a*) Describe all statistical methods, including those used to control for confounding | pp. 12–13 | ITS segmented regression (time trend + level/trend changes at 3 breakpoints); HC1 robust SEs; mixed-effects models |
|  |  | (*b*) Describe any methods used to examine subgroups and interactions | pp. 12–13 | Period-by-time interaction in reviewer-consistency ; document-type-specific analyses |
|  |  | (*c*) Explain how missing data were addressed | p. 10 | No missing discharge date-times (2019–2025) |
|  |  | (*d*) *Cohort study*—If applicable, explain how loss to follow-up was addressed  *Case-control study*—If applicable, explain how matching of cases and controls was addressed  *Cross-sectional study*—If applicable, describe analytical methods taking account of sampling strategy | p. 11; p. 13 | Exclusion of records from reviewers with <10 records; joint-reviewer exclusion and workload ≥100 for consistency analysis |
|  |  | (*e*) Describe any sensitivity analyses | pp. 21–22 | Breakpoint shifts ±1/±2 months; excluding 2020; placebo breakpoint Jan 2020; high-risk threshold 5; workload thresholds ≥50/≥200/≥500 |
| Results | | | | |
| Participants | 13* | (a) Report numbers of individuals at each stage of study—eg numbers potentially eligible, examined for eligibility, confirmed eligible, included in the study, completing follow-up, and analysed | pp. 10–11 | 61,697 retrieved → 61,644 included (53 records excluded) → 43,324 admissions; Figure 5 |
|  |  | (b) Give reasons for non-participation at each stage | pp. 10–11 | 53 records from 28 reviewers with <10 records excluded; joint-reviewer cases excluded for consistency analysis |
|  |  | (c) Consider use of a flow diagram | p. 11 | Figure 5. Participant flow diagram |
| Descriptive data | 14* | (a) Give characteristics of study participants (eg demographic, clinical, social) and information on exposures and potential confounders | p. 11 | Table 1. Annual summary of sampled cases, defect cases, high-risk cases, defect records, and AI write-back |
|  |  | (b) Indicate number of participants with missing data for each variable of interest | p. 10 | Discharge date-times: no missing values |
|  |  | (c) *Cohort study*—Summarise follow-up time (eg, average and total amount) | p. 10 | 84 monthly discharge cohorts (January 2019–December 2025) |
| Outcome data | 15* | *Cohort study*—Report numbers of outcome events or summary measures over time | pp. 14–20 | Table 2 (ITS coefficients); Figures 6–9 (monthly series); Table 3 (consistency) |
|  |  | *Case-control study—*Report numbers in each exposure category, or summary measures of exposure |  |  |
|  |  | *Cross-sectional study—*Report numbers of outcome events or summary measures |  |  |
| Main results | 16 | (*a*) Give unadjusted estimates and, if applicable, confounder-adjusted estimates and their precision (eg, 95% confidence interval). Make clear which confounders were adjusted for and why they were included | pp. 14–18; Table 2 | ITS estimates with 95% CIs; adjusted for time trend and breakpoint level/trend changes |
|  |  | (*b*) Report category boundaries when continuous variables were categorized | - | - |
|  |  | (*c*) If relevant, consider translating estimates of relative risk into absolute risk for a meaningful time period | pp. 15–16 | Counterfactual analyses in absolute percentage points (e.g., defect rate 0.44 percentage points higher by August 2023) |

Continued on next page

| Other analyses | 17 | Report other analyses done—eg analyses of subgroups and interactions, and sensitivity analyses | pp. 17–22 | Stable-rule-set analysis; document-type-specific rates; sensitivity analyses (Tables 5–6) |
| --- | --- | --- | --- | --- |
| Discussion | | | | |
| Key results | 18 | Summarise key results with reference to study objectives | p. 22 | Principal Findings |
| Limitations | 19 | Discuss limitations of the study, taking into account sources of potential bias or imprecision. Discuss both direction and magnitude of any potential bias | pp. 23–24 | Limitations (sampling-source indicator; no direct accuracy evaluation; distinct reviewer cohorts; retrospective design; manual LLM triggering) |
| Interpretation | 20 | Give a cautious overall interpretation of results considering objectives, limitations, multiplicity of analyses, results from similar studies, and other relevant evidence | pp. 22–24 | Interpretation and Comparison with the Literature; Conclusions |
| Generalisability | 21 | Discuss the generalisability (external validity) of the study results | p. 24 | Conclusions and Implications (replicable model; multi-center validation as future work) |
| Other information | |  | | |
| Funding | 22 | Give the source of funding and the role of the funders for the present study and, if applicable, for the original study on which the present article is based | p. 25; p. 13 | Funding (NSFC Grant No. 72204005); Ethics Approval; |

*Give information separately for cases and controls in case-control studies and, if applicable, for exposed and unexposed groups in cohort and cross-sectional studies.

**Note:** An Explanation and Elaboration article discusses each checklist item and gives methodological background and published examples of transparent reporting. The STROBE checklist is best used in conjunction with this article (freely available on the Web sites of PLoS Medicine at http://www.plosmedicine.org/, Annals of Internal Medicine at http://www.annals.org/, and Epidemiology at http://www.epidem.com/). Information on the STROBE Initiative is available at www.strobe-statement.org.
